# Rational Design of a Plasmid Origin That Replicates Efficiently in Both Gram-Positive and Gram-Negative Bacteria

**DOI:** 10.1371/journal.pone.0013244

**Published:** 2010-10-08

**Authors:** Anton V. Bryksin, Ichiro Matsumura

**Affiliations:** Center for Fundamental and Applied Molecular Evolution, Department of Biochemistry, Emory University, Atlanta, Georgia, United States of America; St. Petersburg Pasteur Institute, Russian Federation

## Abstract

**Background:**

Most plasmids replicate only within a particular genus or family.

**Methodology/Principal Findings:**

Here we describe an engineered high copy number expression vector, pBAV1K-T5, that produces varying quantities of active reporter proteins in *Escherichia coli*, *Acinetobacter baylyi* ADP1, *Agrobacterium tumefaciens*, (all Gram-negative), *Streptococcus pneumoniae*, *Leifsonia shinshuensis*, *Peanibacillus* sp. S18-36 and *Bacillus subtilis* (Gram-positive).

**Conclusions/Significance:**

Our results demonstrate the efficiency of pBAV1K-T5 replication in different bacterial species, thereby facilitating the study of proteins that don't fold well in *E. coli* and pathogens not amenable to existing genetic tools.

## Introduction

The laboratory work horse, *E. coli*, cannot efficiently translate or fold many foreign proteins. Most prokaryotic plasmids replicate in a particular eubacterial genus or family. The employment of different bacterial species as expression systems therefore necessitates the acquisition or development of new expression vectors [1], [2]. Broad host range plasmids based on RK2 [3], IncaP [4] or rolling circle replication (RCR) [5] origins have been developed for the production of proteins, or the study of poorly characterized bacterial pathogens, but most are limited in their host range, genetic stability, size or capacity to accept large inserts [2], [6].

pWV01 is a cryptic plasmid originally purified from *Streptococcus cremoris* [7]. Its RCR origin has been used to create over 20 cloning vectors [5]. Among them, pGK12 is most widely used by other researchers. It replicates in *Bacillus subtilis*, *Lactococcus lactis*, *E. coli*, *Borrelia burgdorferi*, and numerous *Lactobacilli* (namely *reuteri*, *fementum*, *casei*, *acidophilus*, *pentosus* and *helveticus*) [6]. Unfortunately, pGK12 is unstable and does not replicate to high copy number in these species. Its performance in *E. coli* is particularly poor, so it was never widely adopted by researchers [6].

Little was known about RCR when pGK12 was first constructed [5]. Three decades of subsequent study [6], [8], [9] have laid the foundation for the rational design of better plasmid origins. RCR plasmids exist in eubacteria (Gram-positive and Gram-negative) and archaeabacteria [6]. Replication begins when the Rep protein, which is encoded on the plasmid (ORF A), recognizes a specific site on the plasmid (double-strand origin, or DSO) and catalyzes the nicking of one DNA strand. The Rep protein remains bound to the 5′ phosphate after the nicking action. The newly released 3′ hydroxyl on the opposite end serves as a primer for DNA synthesis. The host DNA polymerase uses the unnicked circular strand as a template, so that a single replication fork moves around a plasmid until it regenerates the DSO. A second copy of Rep protein catalyzes the cleavage of the newly formed DSO, effectively releasing a single stranded copy of the plasmid. In the absence of Rep, the replication fork continues to move around the template, forming a single stranded concatemer. The single strand origin (SSO), a non-coding element that forms extensive secondary structure, is required for synthesis of the lagging strand. SSO sequences vary considerably among different RCR plasmids, but are extremely important for robust replication of the plasmid in the cell [10]. Here we describe the engineering of the pWV01 RCR origin to create pBAV1K-T5, a very broad-host range expression vector.

## Results and Discussion

We hypothesized that the RCR of pWV01-based plasmids in non-native hosts was inefficient. Cryptic plasmids by definition have no detectable effects on their hosts, so the copy number of pWV01 in its native host must be stringently controlled. If the copy number control mechanisms of a plasmid are more efficient than its RCR mechanisms in non-native hosts, the plamid would not be stable under non-selective conditions. The elimination of regulatory elements, particularly those not widely conserved among RCR plasmids, should allow the altered plasmid to replicate more freely. We sought to create a minimal plasmid origin that included only pWV01 ori elements that were shown to be necessary by other researchers [11]. Runaway replication is toxic to host cells, of course, but we speculated that the inefficiency of RCR in non-native hosts would moderate this risk.

**Figure 1:**
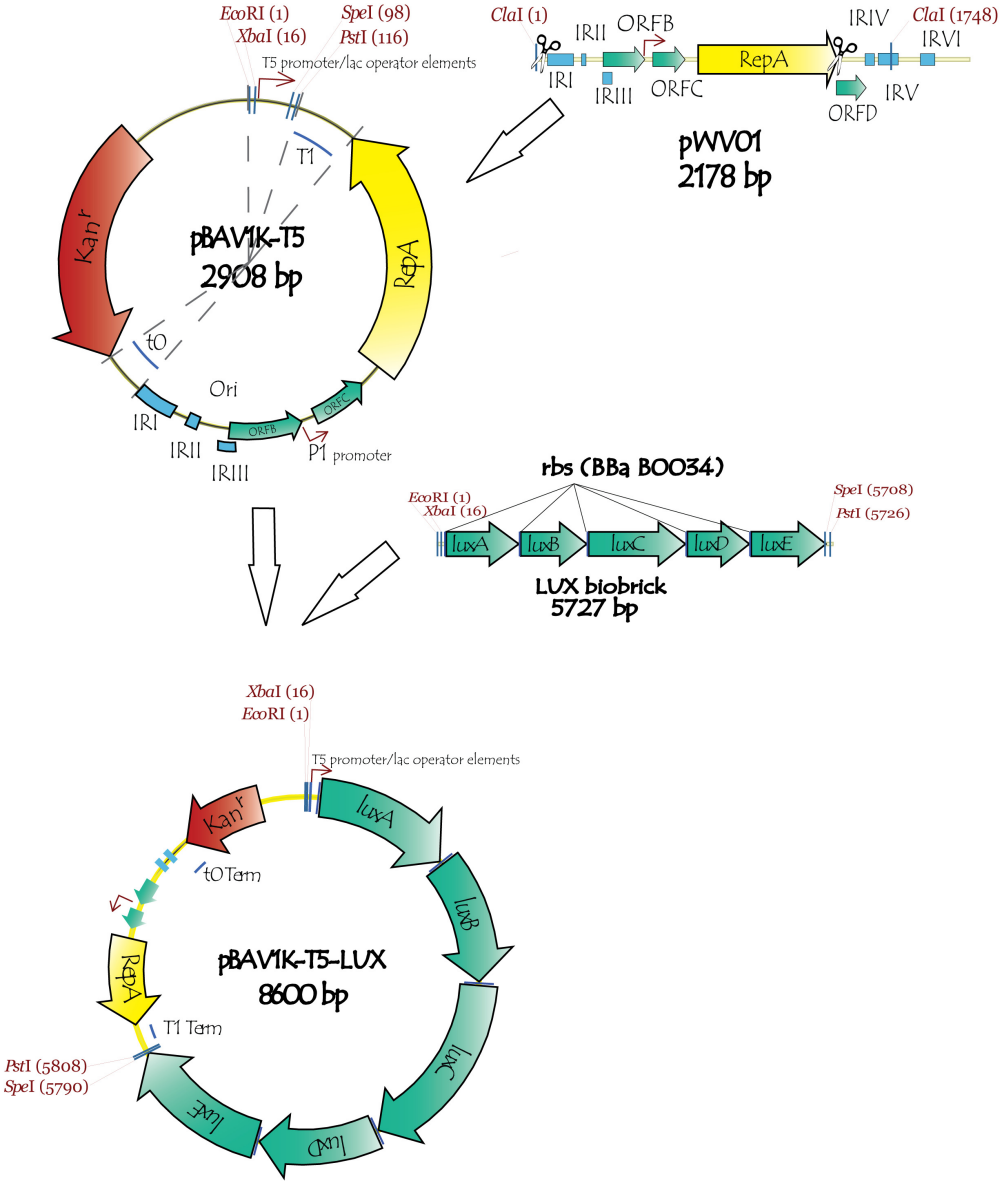
Construction of pBAV1K-T5-*lux*, a very broad host range expression vector. The cryptic plasmid, pWV01, exhibits broad host range but is unstable in many species. The ORF D, and inverted repeats IV, V and VI were deleted from its plasmid origin; terminators t0 and T1 were inserted on opposite ends of the shortened origin (upper right). The selectable marker, the *Enterococcus* 3′,5″-aminoglycoside phosphotransferase type III, and a T5 promoter within a BioBrick multiple cloning site were cloned into the plasmid (top circle). The lux genes of *Photorhabdus luminescens* were individually PCR amplified, cloned, assembled with ribosome binding sites (middle) and cloned into the plasmid to create pBAV1k-T5-*luxABCDE* (bottom circle).

We therefore sought to delete copy number control mechanisms from the pWV01 origin. Two inverted repeats (IRI and IRII), out of the six within the pWV01 origin, are sufficient for the conversion of single-stranded DNA to the double-stranded form [12]. The ORF D protein may play a role in copy number regulation, but is not essential for replication [7]. Three different inverted repeats (IRIV, IRV and IRVI), which may serve as an alternative SSO, and ORF D were deleted (Fig. 1). The ORF C protein is a negative regulator of P1 promoter that regulates the expression of repA [7]; we chose to retain it to moderate the risk of runaway plasmid replication. In addition, the T1 and t0 transcription terminators [13] were inserted on opposite sides of the origin of replication to prevent RNA read-through to the plasmid origin part. The terminators should prevent possible antisense interference by the wild-type RNA transcript with *repA* expression and SSO function.

We completed our expression vector by cloning a selectable marker, a multiple cloning site, a promoter and reporter genes into the plasmid (Fig. 1). Each element was selected for its ability to function in the widest variety of hosts. The 3′,5″-aminoglycoside phosphotransferase type III, under its own promoter, confers kanamycin resistance upon many bacterial species [14]. We introduced a BioBrick multiple cloning site (EcoRI-NotI-XbaI-insert-SpeI-NotI-PstI) so that our vector, pBAV1K-T5, which does not otherwise contain those restriction sites, would be compatible with this standard [15]. We subsequently cloned a BioBrick containing a T5 promoter and two lacO operators [16] into the multiple cloning site (pBAV1K-T5). The *gfp* (0.8 kb), *gusA* (1.8 kb) and *lacZ* (3 kb) reporter genes, and the *luxABCDE* reporter operon (5 kb), were separately cloned downstream of the promoter (pBAV1K-T5-*gfp*, pBAV1K-T5-*gusA*, pBAV1K-T5-*lacZ* and pBAV1K-T5-*lux*).

**Figure 2:**
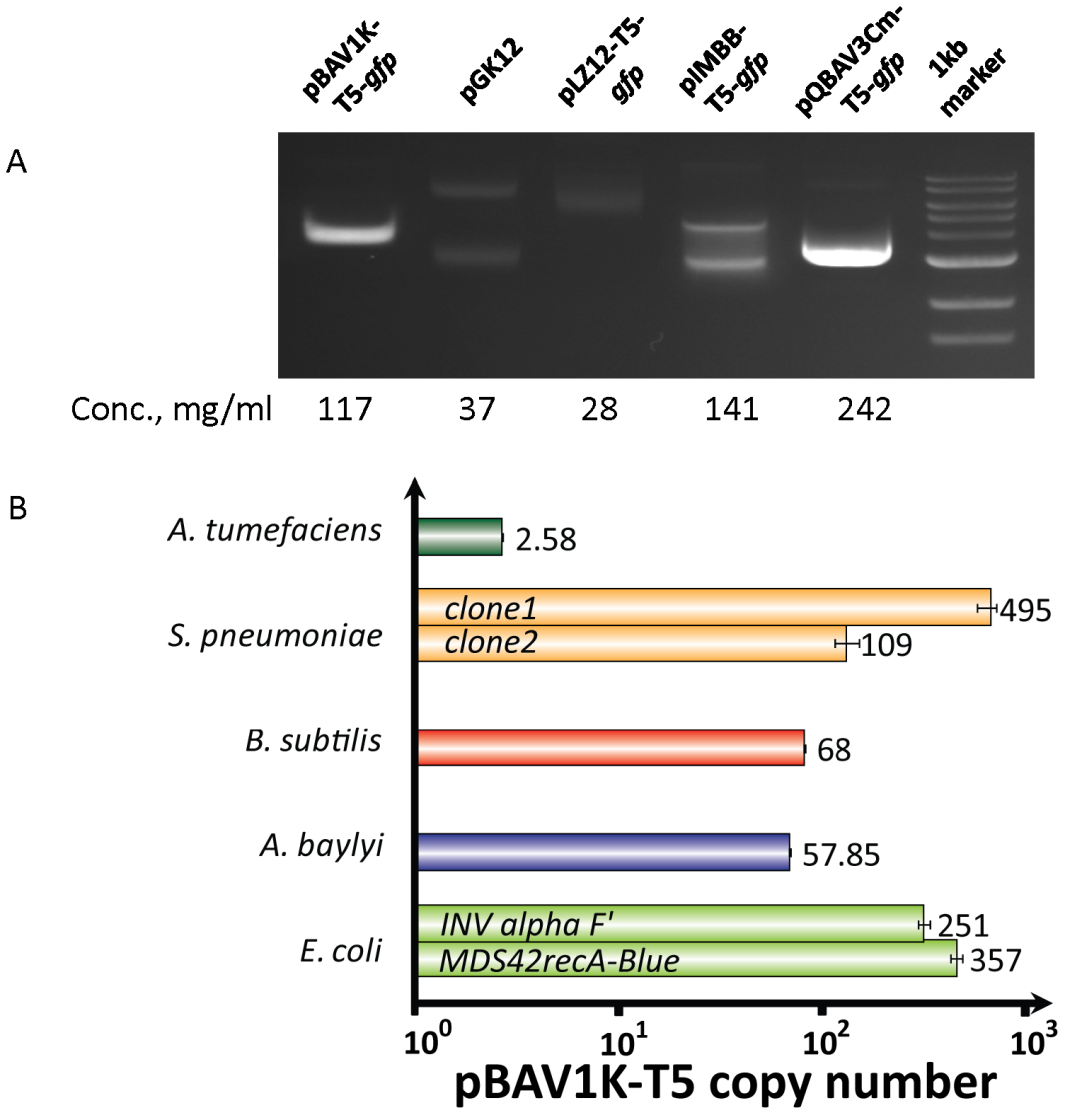
Plasmid pBAV1K-T5-*gfp* replicates to high copy number in *Escherichia coli*. (A) *E. coli* was transformed with plasmids pBAV1K-T5-*gfp*, pLZ12-T5-*gfp*, pGK12 (two other RCR plasmids), pQBAV3Cm-T5-*gfp* or pIMBB-T5-*gfp* (two ColE1 derived plasmids). The transformants were propagated in liquid LB cultures supplemented with the appropriate antibiotics. The plasmids were purified, and 2 microliters of each were analyzed on a 0.8% agarose gel. The higher yield and faster mobility of the pBAV1k relative to the larger pWV01 derivatives indicates supercoiling. (B) Five different species of bacteria (namely *Agrobacterium tumefaciens*, *Streptococcus pneumoniae*, *Bacillus subtilis*, *Acinetobacter baylyi* ADP1 and *E. coli*) were transformed with pBAV1K-T5-*luxABCDE* (Table 3). The APH(3′)-IIIa gene present on the plasmid was used as a target to estimate the copy number in reference to the chromosomal *relA*/*spoT* gene (or its homolog) by quantitative real-time PCR. Each bar represents the average of three replicates. Error bars represent standard error.

Like most other molecular biologists, we use *E. coli* as a cloning vehicle, even if the final construct intended to be used in other species. We transformed *E. coli* with pBAV1K-T5-*gfp*, or control RCR plasmids pGK12 (derived from pWV01) and pLZ12-T5-*gfp* (derived from pSH71[17] and has high level of homology to pWV01) or ColE1 plasmids (pQBAV, or pIMBB). The transformants were propagated in liquid LB cultures and lysed. The yield of plasmid from the pBAV1K-T5-*gfp* transformant was comparable to those of the ColE1-derived plasmids (Fig. 2a). The visibly higher amount of purified vector for pBAV1K-T5-*gfp*, relative to parental plasmids pGK12 and pLZ12-T5-*gfp*, was confirmed by Real-Time qPCR analysis(Fig. 2b). pBAV1K-T5-*gfp* replicates to 357 copies per cell in MDS42recA-Blue and 251 copies per cell in INV alpha F′. In contrast, pGK12 replicated only to 60 copies/cell in *E. coli* [5].

**Figure 3:**
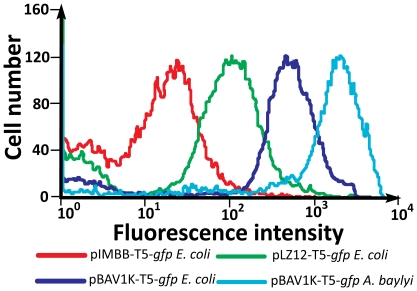
Heterologous GFP expression from pBAV1K-T5-*gfp*, pLZ12-T5-*gfp* and pIMBB-T5-*gfp*. *E. coli* was transformed with pIMBB-T5-*gfp*, pLZ12-T5-*gfp*, or pBAV1k-T5-*gfp*. *A. baylyi* was transformed with pBAV1kT5-*gfp* only. The transformants were propagated in liquid LB supplemented with the appropriate antibiotic and diluted to 5×10^5^ cells per ml with M9 minimal media before flow cytometric analysis. The major peaks indicate differences in GFP expression; the minor peaks (left) indicate cells that have don't express GFP, possibly due to plasmid instability or cell death.

We were concerned about the effect of high plasmid copy number (runaway replication) on cell fitness, so we used flow cytometry to assess the genetic stability of the plasmid of the transformed cells. We created T5-*lacO-lacO-gfp* versions of our vectors (pBAV1K-T5-*gfp*, pLZ12-T5-*gfp*, pIMBB-T5-*gfp*, and pQE30-T5-*gfp*) in parallel, using overlap extension PCR cloning (Materials and Methods). It proved far easier to clone genes into pBAV1K-T5 than into the other RCR-replicating plasmids (pLZ12 and pGK12). When *E. coli* were transformed with the *in vitro* recombination reactions, 172 pBAV1K-T5-*gfp* transformant colonies formed. In contrast, a single pLZ12-T5-*gfp* transformant colony grew on the second try, and no pGK12-T5-*gfp* transformants grew in four attempts. We hypothesized that the poor performance of pLZ12 and pGK12 as cloning vectors reflected their inability to replicate stably in *E. coli*. Indeed, flow cytometry analysis of mid-log (OD600 = 0.8) showed that populations of *E. coli* cells transformed with pBAV1K-T5-*gfp* had much lower proportions of non-fluorescent (and potentially dead) cells than populations of isogenic pLZ12-T5-*gfp* transformants (Fig. 3).

**Figure 4:**
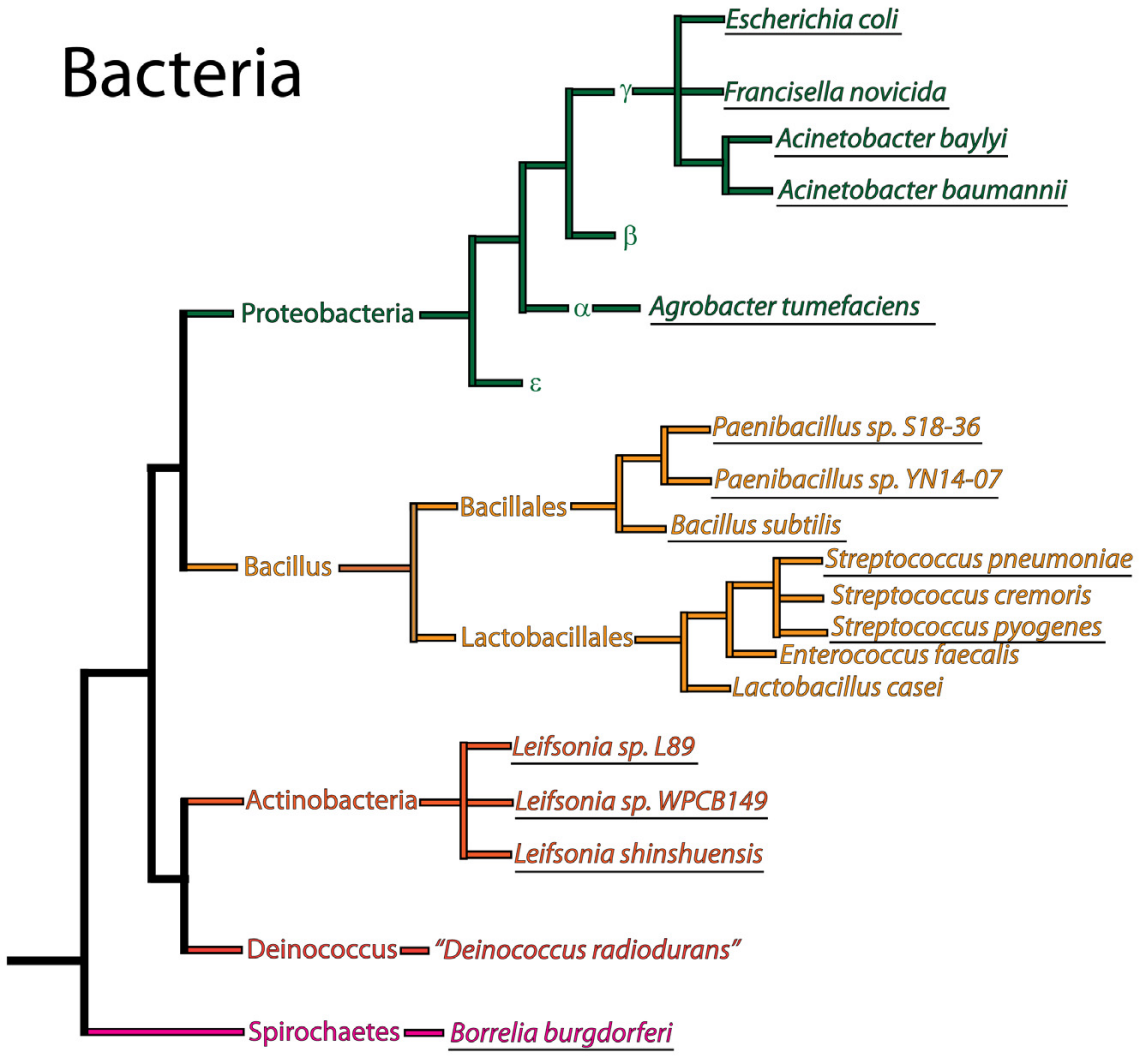
Dendrogram of bacterial species. Dendrogram depicting the phylogenetic relationships among eubacteria adapted from phylogenetic trees inferred by Jun et al. [25] Cheng et al. [26] and Brown et al. [27]. Branch lengths do not represent evolutionary distance. Only species related to the study are shown; those that that have demonstrated the capacity to harbor the pBAV1K-T5-*gfp* and/or pBAV1k-T5-*luxABCDE* plasmid are underlined.

**Figure 5:**
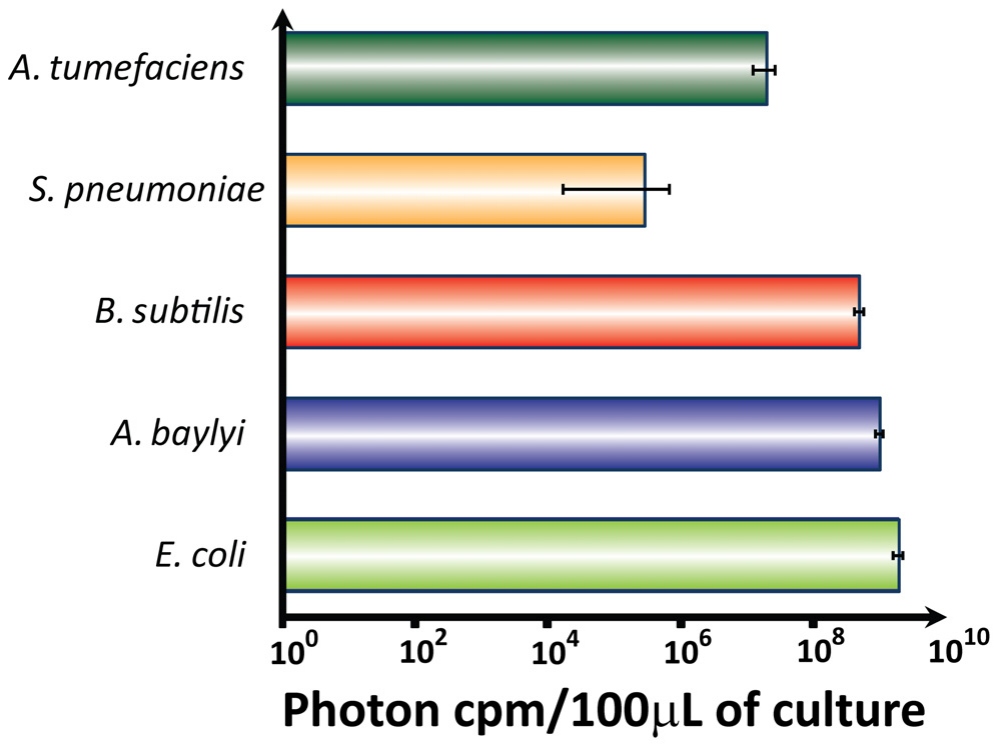
Heterologous expression of *lux* operon from pBAV1K-T5-*luxABCDE*. Five bacterial species (Fig. 2) were transformed with pBAV1K-T5-*lux*. The transformants were propagated in liquid culture (Table 3). The luminescence from equal volumes of culture (100 microliters) was measured in a luminometer. The values (plotted on a log scale) represent the averages of three independent experiments.

pBAV1K-T5-*luxABCDE* plasmid DNA purified from *E. coli* was used to transform *E. coli*, *A. baylyi*, *S. pneumoniae*, *A. tumefaciens and B. subtilis* (underlined in Fig. 4). Every species we tested was successfully transformed, except *Deinococcus radiodurans*, possibly because this species harbors its own incompatible cryptic plasmid [18]. The transformants were propagated in rich media, and the luminescence of 100 microliters of liquid culture was measured. Each species produced light, confirming the broad host specificity of the promoters, ribosome binding sites and selectable markers in our vector (Fig. 5). Light production is a function of many factors, including the plasmid copy number, codon bias, protein folding and the metabolic network of each host species, so the variations between species are difficult to rationalize. Plasmid DNA copy number in *E. coli*, *A. baylyi*, *B. subtilis* and *A. tumefaciens* was measured by real-time quantitative PCR using whole DNA purified from the bacterial cells; a relative quantification was used for copy number calculation (Fig. 2b). In *B. subtilis*, pBAV1K-T5-*luxABCDE* replicates to 10 times the reported copy number of pGK12 in the same species [5].

**Figure 6:**
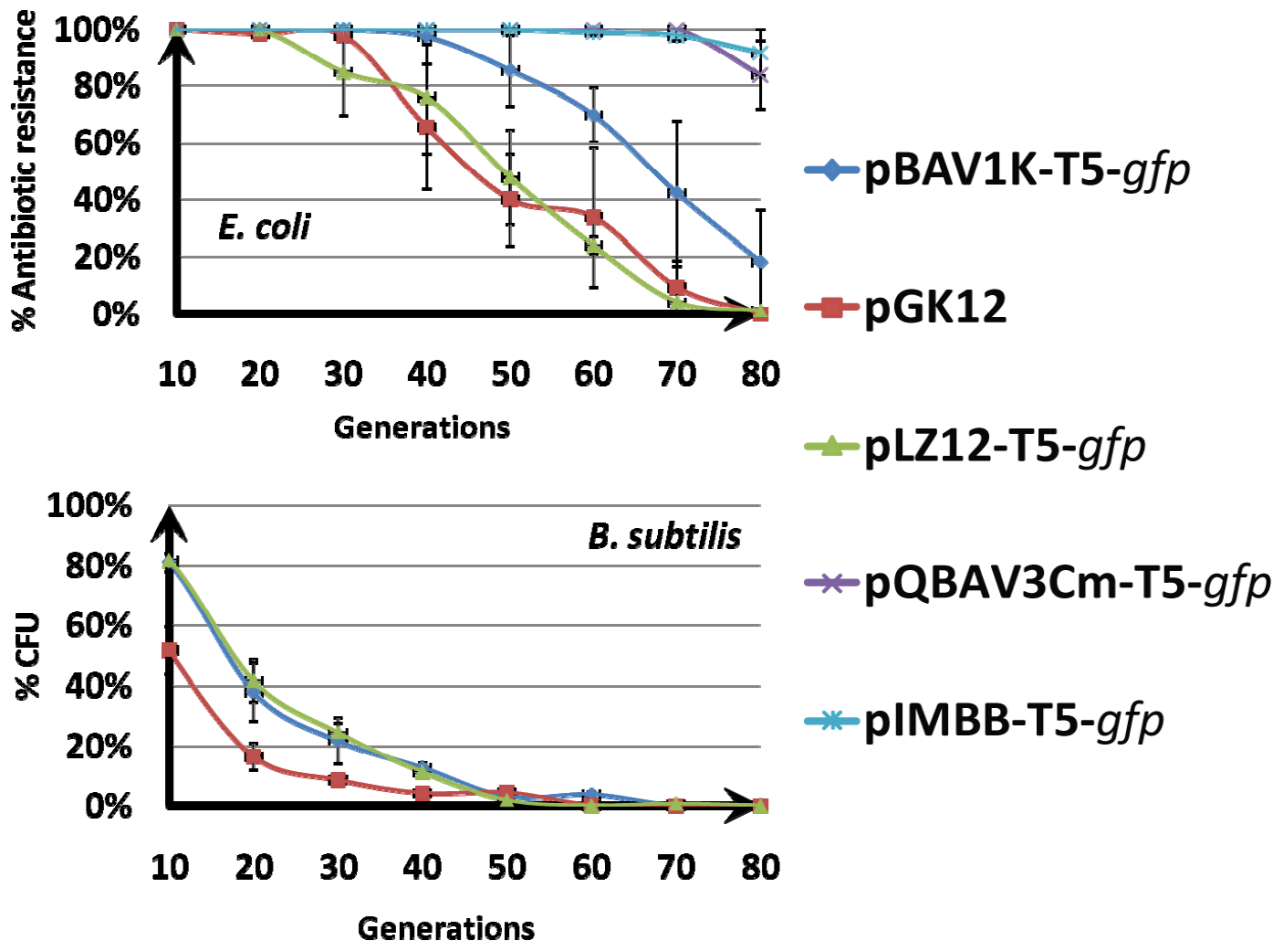
Plasmid stability assays. (A) Plasmids pBAV1K-T5-*gfp*, pGK12, pLZ12-T5-*gfp*, pQBAV3Cm-T5-*gfp* and pIMBB-T5-*gfp* were separately propagated in *E. coli* for 80 generations without antibiotic selection. Plasmid stability was determined by replica plating onto selective media and presented as a percentage of cells that retain antibiotic resistance. (B) Plasmids pBAV1K-T5-*gfp*, pGK12, pLZ12-T5-*gfp*, were propagated in *B. subtilis* for 80 generations without antibiotic selection. Plasmid stability was determined by the plasmid content comparison in the total DNA pools between different generations. Error bars represent standard error.

All bacterial cells transformed with pBAV1K-T5-*gfp* retain the plasmid when propagated under selective conditions (i.e. in media containing kanamycin). To determine whether the vector could stably replicate under non-selective conditions, *E. coli*, and *B. subtilis* were separately transformed with pBAV1K-T5-*gfp*, pGK12, or pLZ12-T5-*gfp*. *E. coli* cells were also separately transformed with pIMBB-T5-*gfp* or pQBAV3Cm-T5-*gfp* in parallel. The transformants were separately grown in non-selective broth for 80 generations by serial dilution and agitation, and the percentage of antibiotic-resistant colonies in the total viable count was determined. In *E. coli*, plasmids pBAV1K-T5-*gfp* (approximately 18% kanamycin-resistant colonies after 80 generations) exhibited higher stability than either pLZ12-T5-*gfp* or pGK12 (0% chloramphenicol-resistant colonies after 80 generations, Fig. 6). The specialized ColE1 based plasmids were, however, more stable than pBAV1K-T5-*gfp* in *E. coli* (80% and 90% ampicillin-resistant colonies after 80 generation for pQBAV3Cm-T5-*gfp* and pIMBB-T5-*gfp* respectively). When *B. subtilis* transformants were cultured for 80 generations in non-selective broth, pBAV1K-T5-*gfp* and pLZ12-T5-*gfp* exhibited greater stability than pGK12 (Fig. 6b). It is possible that the expression of the erythromycin resistance marker from pGK12 plasmid increased the cost of the plasmid in nonselective conditions, resulting in the earlier elimination of the plasmid from the bacterial population.

The pBAV1K-T5-*gfp* expression vector was also used to transform natural bacterial species in two arbitrarily collected soil samples. These experiments extended the known host-specificityof the plasmid and revealed naturally competent bacterial species. Gene transfer by natural transformation allows bacteria to adapt rapidly to changing environmental conditions. It probably occurs all the time, but is too infrequent to detect under natural conditions, particularly in soil [19], [20], [21]. The soil was mixed with the aqueous sample of the plasmid, agitated overnight at room temperature and allowed to grow in rich medium supplemented with kanamycin. Fluorescent colonies formed on the plates were collected and later identified by 16S RNA gene sequencing as *Peanibacillus* sp. S18-36 and YN14-0; *Leifsonia* sp.L89, WPCB149 and *shinshuensis*. The pBAV1K-T5 vector will allow researchers to study natural transformation, and evolution, of the soil bacteria. These species are unrelated to the others that we transformed (*E. coli*, *A. baylyi*, *S. pneumoniae*, *A. tumefaciens*, *B. subtilis*, *B. burgdorferi*, *A. tumefacienes*, Fig. 4). We have also shared the vector with collaborators, and have heard that it also replicates efficiently in *A. baumannii*, *S. pyogenes* and *F. novicida* (personal communication, Justin Gallivan, Julia Bugrysheva and David S. Weiss). We are therefore optimistic that pBAV1K-T5 will function in many other mesophilic eubacteriaThe genetic features of the natural occurring plasmids, including promoters, ribosomal binding sites, terminators, and codon usage, typically coincide with those of their hosts. The plasmid must serve as a template for host replication factors, namely DNA gyrase, DNA ligase, RNA polymerase, DNA polymerase I, and DNA polymerase III; the host ribosome must recognize the ribosome binding site of the *repA* transcript. Eubacterial genomes have been diverging from their last common ancestor for over three billion years. We were therefore surprised that pBAV1K-T5 replicated in so many species (Fig. 4), imparting kanamycin resistance and reporter protein production upon each. The factors that mediate transcription and translation in these Gram-negative and Gram-positive species must therefore be conserved to a heretofore underappreciated extent.

We intend to distribute pBAV1k-T5 without regard to intellectual property considerations. Microbiologists could employ it as genetic tool to elucidate the pathogenic mechanisms of poorly characterized bacteria. Protein engineers could use it to express their favorite genes in a variety of eubacteria. It is difficult to predict *a priori* which species will produce the highest yield of any particular protein, but we showed here that *E. coli* is not always the best choice. Synthetic biologists could better leverage the complex molecular machines that already exist within the vast domain of the eubacteria.

## Materials and Methods

### Materials

All primers used in the study (Table 1) were purchased from IDT (Coralville, IA). All plasmids (Table 2) and bacterial strains (Table 3) were obtained from commercial vendors or collaborators. Restriction enzymes and DNA modification enzymes were from NEB (Ipswich, MA), and reactions were carried out under the recommended conditions. All other chemicals used in the study were from Sigma-Aldrich (molecular biology grade). Plasmid DNA from *E. coli* was purified with the QIAprep spin miniprep kit as directed by the manufacturer (QIAGEN, Valencia, CA).

**Table 1:** Oligonucleotides and PCR primers used in the study.

Primer	Sequence 5′-3′	Reference
AVB 5	TTCTTAGACGTCAAATTCTATCATAATTGTGGTTTCAAAATCGGCTCCGTCG	[28]
AVB6	ACTGGATCTATCAACAGGAGTCCAAGCGAGCTCGGTACTAAAACAATTCATCCAGTAAA	[28]
AVB25	ATAGAATTTGACGTCTAAGAAACCATTATTATCATGACATTAACC	This study
AVB26	GACTGAGCCTTTCGTTTTATTTGATGCCTCTAGATTAATTAATTAAGCGGCCGCATCGATCG	This study
AVB29	CGATTTTTTATTAAAACGTCTCAAAATCG	This study
AVB30	GTCATTTTATTTCCCCCGTTTCAGCA	This study
AVB 33	GGTTGCCGCCGGGCGTTTTTTATTGGTGAGAATCCAAGCACTAGGCGATTTTTTATTAAAACGTCTCAAAATCG	This study
AVB34	TACCGAGCTCGCTTGGACTCCTGTTGATAGATCCAGTAATGACCTCAGAA	This study
AVB35	CAACAAACTCTAGCGCCTTTAGATTATGGTTTGAGGGCAATTATCAGTGTGGATATAGAGCAAGTTATGCAAAGGTTCTTGA	This study
AVB36	CCTACTCAGGAGAGCGTTCACCGACAAACAACAGATAAAACGAAAGGCCCAGTCTTTCGACTGAGCCTTTCGTTTTATTT	This study
AVB37	TGTCGGTGAACGCTCTCCTGAGTAGGACAAATCCGCCGCCCTAGACCTAGTGTCATTTTATTTCCCCCGTTTCAGCA	This study
AVB38	ATTAATCTAGAGGCATCAAATAAAACGAAAGGCTCAGTCGAAAGAC	This study
AVB199	GGATCCCTGCAGGCCTCAGGGCCCGATCGATGCCGCCGCTTAATTAATTAATCCAGAGGC	This study
AVB200	CTAGAAGCGGCCGCGAATTCGACGTCAAATTCTATCATAATTGTGGTTTCAAAATCGGC	This study
AVB221	AATTCGCGGCCGCTTCTAGAGGAAATCATAAAAAATTTATTTGCTTTGTGAGCGGATAACAATTATAATAGATTCAATTGTGAGCGGATAACAATTA	This study
AVB222	CTAGTAATTGTTATCCGCTCACAATTGAATCTATTATAATTGTTATCCGCTCACAAAGCAAATAAATTTTTTATGATTTCCTCTAGAAGCGGCCGCG	This study
AVB264	GGTCGTCAGACTGATGGGCCCCTGCATCAGGGCGATGGCCCACTACGTGG	This study
AVB265	GGCACAGATGGTCATAACCTGAAGGAAGATCTGGGGCCTTTTGCTGGCCTTTTGCTCACATG	This study
AVB270	GCCGATTTTGAAACCACAATTATGATAGAATTTGACGTCATCAGGGCGATGGCCCACTACGTGG	This study
AVB271	AGCGGCGGCATCGATCGGGCCCTGAGGCGGCCTTTTGCTGGCCTTTTGCTCACATG	This study

**Table 2:** Plasmids used in the study.

Plasmid	Description	Reference
pGK12	pWV01 ori; Erm^r^ Cam^r^	[5]
pLZ12	Modified pNZ12 vector; pSH71 ori (high level of similarity to pWV01); Cam^r^	[29].
pLZ12-T5-*gfp*	T5lacO; Cam^r^	This study
pBAV1K-T5	T5lacO; Kan^r^	This study
pBAV1K-T5-*gfp*	T5lacO; Kan^r^	This study
pBAV1K-T5-*gus*	T5lacO; Kan^r^	This study
pBAV1K-T5-*lacZ*	T5lacO; Kan^r^	This study
pBAV1K-T5-*luxABCDE*	T5lacO; Kan^r^	This study
pIMBB-T5-*gfp*	Custom biobrick accepting vector; T5lacO; ColE1 replicon; Amp^r^	[22]
pIMBB	Custom biobrick accepting vector; ColE1 replicon; Amp^r^	[22]
pQBAV3Cm-T5-*gfp*	Vector based on pQE30 (Qiagen) ; T5lacO; ColE1 replicon; Cam^r^;	[22]
pQBAV3Cm-T5-*gusA*	Vector based on pQE30 (Qiagen); T5lacO; ColE1 replicon; Cam^r^;	[22]
pQBAV3Cm-T5-*lacZ*	Vector based on pQE30 (Qiagen); T5lacO; ColE1 replicon; Cam^r^;	[22]
pQBAV3Cm-T5-*luxABCDE*	Vector based on pQE30 (Qiagen); T5lacpo; ColE1 replicon; Cam^r^; *lux* genes are from *Photorhabdus luminescens* (ATCC number 29999)	[22]

**Table 3:** Bacterial strains and transformation procedures used in the study.

Bacterial Strain	Description	Growth conditions	Transformation protocol	Kan [C, mkg/ml] for plasmid selection	Reference or source
***E. coli***					
MDS42 recA Blue	15% reduced genome size	LB, 37°C	Chemically competent cells	50	Scarab Genomics [30].
INV alpha F′	F′ endA1 recA1 hsdR17 (rk−, mk+) supE44 thi-1 gyrA96 relA1 ϕ80lacZΔM15 Δ(lacZYA-argF)U169 λ-	LB, 37°C	Chemically competent cells	50	Invitrogen
***B. burgdorferi***					
B31	high passage, non-pathogenic strain	BSK-II, 34°C	Electroporation [31]	400	[31]
***A. tumefaciens***					
C58		LB, 28°C	Electroporationl [32];	50	[33]
***B. subtilis***					
JH642		LB, 37°C	Naturally competent cells [34]	20	[35]
***S. pneumoniae***					
R6	non-encapsulated derivative of the serotype 2 strain D39	Todd-Hewitt medium containing 0.5% yeast extract, 37°C, CO_2_	Naturally competent [36]	400	[37]
***A. baylyi***					
ADP1		LB, 30°C	Naturally competent cells [38]	50	[38]
***F. novicida***					
U112	Low virulence strain	Tryptic Soy Broth supplemented with 0.2% L-cysteine	Chemical transformation [39]	30	[39]

### Recombinant DNA

We used Overlap Extension PCR cloning [22] or restriction enzymes and ligase, to create recombinant plasmids; BioBricks were assembled as directed in the BioBrick Assembly Manual (NEB, Ipswich, MA). When necessary, DNA fragments were purified from agarose gels with QIAquick-gel extraction kits from QIAGEN (Valencia, CA). DNA fragments and PCR mixtures were analyzed on 0.8% Seakem LE agarose gels (Lonza Rockland, Rockland, ME) using the 1 kb DNA ladder (New England BioLabs, Ipswich, MA) a molecular size standard. DNA was sequenced by Macrogen (Rockville, MD).We propagated and transformed established laboratory bacteria according to published procedures (Table 3).

### Transformation and identification of bacteria from soil samples

The soil samples (1 g each) were each mixed with water (100 microliters) and pBAV1K-T5-*gfp* (10 micrograms) and agitated overnight at room temperature. The sample was diluted with LB-kanamycin (1 mL, 100 microgram/mL), agitated for another two hours and spread on LB agar plates supplemented with kanamycin.. After several days of incubation at room temperature, transformed, fluorescent colonies were picked and propagated in liquid LB-kanamycin. Total DNA was prepared with the DNeasy kit (as directed by the Qiagen for gram-positive bacteria). The 16S RNA genes were PCR amplified (with the primers 27F and 1492R [23] ) and sequenced with the same primers and two others (946F and 518R [23]).

### Construction of pBAV1K-T5 and its derivatives

The BioBrick accepting vector pBAV1K-T5 was created by combining individual parts of the vector (Fig. 1) by overlap extension PCR [24]. First, the part of pWV01 origin of replication from IRI to the end of *repA* gene was PCR amplified using primers AVB29 and AVB30 (Table 1). The T1 and t0 *E. coli* transcription terminators were synthesized by overlap extension PCR reaction of primers AVB33, AVB34, AVB35 (t0) and AVB36, AVB37, AVB38 (T1). Second, the PCR products were then combined in another overlap extension PCR reaction that produced the shortened pWV01 origin of replication flanked by transcriptional terminators. Third, overlap extension PCR was used to combine obtained DNA fragment with the kanamycin resistance marker and the tetracycline promoter/multiple cloning site. The kanamycin resistance marker, the APH(3′) (5″)-IIIa gene from *Enterococcus faecalis*, was PCR amplified with its own native promoter using primers AVB5 and AVB6. To obtain the pBAV1K-T5 vector, the synthetic T5 promoter/lac operator [16] was created by overlap extension PCR of primers AVB221 and AVB222 and cloned into custom biobrick accepting vector pIMBB [22]. The plasmid intermediate (engineered origin plus APH(3′)(5″)-III a) was PCR amplified with the primers AVB199 and AVB200. The PCR product, and the T5 promoter/lac operator biobrick, were digested with *EcoR*I and *Pst*I restriction endonucleases and ligated to each other.

Different custom biobricks *luxABCDE* operon, *gfp*, *gusA*, *lacZ* were when cloned into pBAV1K-T5 vector by standard methods [13] or overlap extension PCR cloning [22]. Primers AVB270 and 271 were used to transfer biobricks through overlap extension PCR cloning from pIMBB into pBAV1K-T5. The pBAV1KT5-*gfp* construct was sequenced. The annotated sequence was submitted to GenBank (http://www.ncbi.nlm.nih.gov/genbank/; accession number HQ191434). We intend to distribute our expression vector pBAV1KT5-*gfp* through Addgene plasmid repository (addgene.org) and ATCC (http://www.atcc.org/). The pLZ12-T5-*gfp* was made from pLZ12 by overlap extension PCR cloning with primers AVB264 and AVB265. T5-*gfp* biobrick was transferred from pIMBB-T5-*gfp* to pLZ12 using primers AVB264 and AVB265.

### Plasmid stability test

Cultures of *E. coli* cells, each harboring a plasmid (pBAV1K-T5-*gfp*, pGK12, pLZ12-T5-*gfp*, pQBAV3Cm-T5-*gfp* or pIMBB-T5-*gfp*, Fig. 6A) were propagated in LB medium containing the appropriate antibiotic at 37°C from a single colony. An aliquot (10 microliters) of each overnight culture was inoculated in 10 milliliters of fresh LB medium without antibiotic and grown for 24 hrs until the cells reached stationary phase. At this point the OD_600_ of the culture was typically between 2.0–2.4, which corresponds to a titer of ∼2×10^9^ cells/milliliter. An aliquot (100 microliters) of this stationary phase culture was used to inoculate 100 milliliters of fresh medium and this process of sub-culturing was repeated for eight days. Since each inoculum was 0.1% (100 microliters in 100 ml) it represented a 1000 fold increase in the cell number. The OD and cfu/milliliter values were similar at the end of each round of sub-culturing. Thus every 24 hr period of growth represents 10 doublings and 10 generations. The fraction of untransformed cells emerging within each culture was calculated by plating appropriate dilutions of this culture onto LB plates (without antibiotics) to get isolated colonies. From each LB agar plate, 96 colonies were transferred onto 96-well plates containing LB plus antibiotic; the fractions of plasmid containing cells were calculated by counting the number of wells that had visible bacterial growth.

*B. subtilis* cells harboring the plasmid were similarly grown from a single colony in LB medium containing the appropriate antibiotic at 37°C. An aliquot of 10 microliters of this overnight culture was inoculated in 10 milliliters of fresh LB medium without antibiotic and grown for 24 hours until the cells reached stationary phase. At this point the OD_600_ of the culture was typically between 1.0–1.3. An aliquot (100 microliters) of this stationary phase culture was used to inoculate 100 milliliters of fresh medium and this process of sub-culturing was repeated for eight days. To avoid fails-positive results due to integration of the plasmid into the chromosome of *B. subtilis*, a method different from that used for *E. coli* was used to calculate the percentage of *B. subtilis* cells containing the plasmid. Total DNA was purified from 2 mL aliquots of each overnight saturated culture with Qiagen DNeasy kit. The eluate (1 microliters out of a 250 microliter elution volume) was used to transform chemically competent *E. coli* INV alpha F′ cells. The transformants were spread on LB plates supplemented with appropriate antibiotic; the colonies (corresponding to different generations of the *B. subtilis* culture) were counted. The number of the colonies obtained with the generation 0 culture was considered 100%. Test plasmid purifications and agarose gel electrophoresis were performed from several colonies to confirm plasmids identities.

### Real-Time PCR assays

The copy numbers of the pBAV1K-T5-*luxABCDE* within different cell types were assessed by real-time PCR. Amplification and detection were carried out in LightCycler® 480 (Roche) using sequence specific fluorescent probes from “Universal ProbeLibrary®” (Roche). PCR primers were designed using Primer3 software located at the “Assay Design Center” of the Roche web-site. Total DNA from bacterial species was purified with Qiagen DNeasy kit (as directed by the manufacturer for gram-positive bacteria) and quantified in a ThermoFisher Nanodrop spectrophotometer. The ACIAD3326 (*relA*/*spoT* homolog), a single-copy gene on the chromosome of *A. baylyi*, and orthologues in other bacterial species, were used as a references (genes, primers and probes are listed in Table 4). A 59 bp fragment of the gene was amplified with the primers AVB 126 and AVB 127; probe #47 from Universal ProbLibrary (Roche) was used for detection of the product. The APH(3′)-IIIa gene was used as a target to estimate the copy number for the vector. A 59 bp fragment of the APH(3′)-IIIa gene was amplified with the primers AVB 128 and AVB 129; probe #48 from Universal ProbLibrary® (Roche) was used to detect the product. Both target and reference DNA standards were diluted in 8 to12 serial steps, each applied in duplicate. LightCycler® 480 Probes Master was used for the preparation of all samples. The PCR conditions included a single denaturation cycle of 95°C for 7 min, followed by 45 cycles of 95°C for 10 s, and combined annealing- elongation for 1min at 55°C. All real-time PCRs were done in triplicate and average results are reported.

**Table 4:** Probes and Primers for Real Time PCR used in the study.

Primer	Bacterial specie/Target gene	Sequence 5′-3′	Probe
AVB 126	*A. baylyi*/ACIAD3326	CGCAGACCGCTATCATAACA	#47
AVB 127	*A. baylyi*/ACIAD3326	CGTGCACGTTTGTCTGGT	#47
AVB 128	/(APH)(3′)(5″)-IIIa	GCGCGGATCTTTAAATGG	#48
AVB 129	/(APH)(3′)(5″)-IIIa	GATCTGGCCGATGTGGATT	#48
AVB 283	*E. coli*/spoT (ECBD_0075)	CTGGTAGCCACGGATATTACG	#138
AVB 284	*E. coli*/spoT (ECBD_0075)	AGCCCCGGTAAAGGTCTG	#138
AVB 285	*B. subtilis/relA*	CCCACTCTACCGGGATCAG	#117
AVB 286	*B. subtilis/relA*	CGAAGACTGTCCGAATGTCA	#117
AVB 287	*A. tumefaciens*/NP_354053.2	GCATCGGCACTGACTTCTC	#42
AVB 288	*A. tumefaciens*/NP_354053.2	TTCAGTTGCCGCAAATCC	#42
AVB 289	*Streptococcus pneumoniae/spoT*	GAAAGACAAGTCTTCTAATTCCCATT	#47
AVB 290	*Streptococcus pneumoniae/spoT*	GAAATCTATGCCCCACTTGC	#47

### Bacterial detection GFP+/GFP- discrimination by flow cytometry

*E. coli* INV alpha F′ cells transformed with pBAV1K-T5-*gfp*, pLZ12-T5-*gfp* or pIMBB-T5-*gfp* were grown in LB at 30°C with aeration to the OD_600_ = 1.2. *A. baylyi* ADP1 cells were grown in LB at 30°C with aeration to the OD_600_ = 0.8. Cells were then washed twice with M9 minimal media, resuspended in M9, and analyzed by flow cytometry with a FACSCalibur flow cytometer (BD Biosciences; San Jose, California). Cell samples were diluted to approximately 5×10^5^ cells per ml with M9 minimal media and delivered at the flow rate of 50 to 150 cells/sec. The FSC (Forward Scatter), SSC (Side Scatter), and fluorescence signal were measured. A band pass filter of 530 nm (515 to 545 nm) was used to collect the green fluorescence. All signals were collected by using logarithmic amplifications. A combination of FSC and SSC were used to discriminate bacteria from background. A total of 20,000 events for each sample were collected and analyzed with the CellQuest Pro software.

### Luminescence assay

*E. coli* MDS42 recA, *A. tumefaciens*, *B subtilis* and *A baylyi* were transformed with pBAV1K-T5-*luxABCDE* vector. The transformed cells, and untransformed negative controls were grown in rich media to mid-exponential phase with aeration. *S. pneumoniae* cells were grown in Todd-Hewitt medium containing 0.5% yeast extract without aeration but were aerated for 2 hours at room temperature before measurement. After incubation, luminescence from 100 microliters of each bacterial culture was measured with a SpectraMax M5 Multi-Mode Microplate Reader (Molecular Devices; Sunnyvale, CA). All the measurements were performed in triplicate independent experiments, each in octuplicate.
